# Determining the utility of a screening program to reduce the incidence of HPV driven oropharyngeal cancer

**DOI:** 10.18632/oncoscience.541

**Published:** 2021-08-09

**Authors:** Sarju Vasani, Ian Frazer, Chamindie Punyadeera

**Affiliations:** ^1^Department of Otolaryngology, Royal Brisbane and Women’s Hospital, Queensland, Australia; ^2^Faculty of Medicine, The University of Queensland, Queensland, Australia; ^3^Translational Research Institute, Queensland, Australia; ^4^Saliva & Liquid Biopsy Translational Laboratory, School of Biomedical Sciences, Faculty of Health, Queensland University of Technology (QUT), Queensland, Australia

**Keywords:** oropharyngeal cancer, human papillomavirus, screening, early detection, saliva

## Abstract

The last decade has seen a continued escalation in rates of human papillomavirus related oropharyngeal malignancy (HPV-OPC). This has occurred despite established national vaccination programs. In contrast, HPV associated cervical cancer incidence rates have declined, due in part to effective cervical cancer screening programs, many of which have moved towards the detection of high-risk HPV (hrHPV) as an early marker of malignant potential. This raises questions as to whether similar hrHPV screening methods could be used for early detection of HPV-OPC. Persistent oral hrHPV is a prerequisite for the development of HPV-OPC and can be accurately detected in saliva. Despite this, single point saliva testing for hrHPV lacks sufficient sensitivity and specificity to allow for effective population screening. Recent published literature suggests the use of serial saliva testing in targeted high-risk individuals, with an emphasis on biomarker persistence and intensity patterns, as a potential means of detecting even subclinical microscopic disease. When coupled with serological testing, this has the potential to provide an accurate test for screening at risk individuals. Despite these promising developments, several significant barriers to an effective targeted screening program remain.

## INTRODUCTION

Rates of human papillomavirus (HPV) related oropharyngeal malignancy (HPV-OPC) are increasing rapidly throughout high-income countries. HPV-OPC numbers have now surpassed those of cervical cancer, making it the most common HPV related malignancy in many nations. Risk factors for the disease include male sex, current smoking status, and a greater number of lifetime sexual partners. Vaccination programs aimed at eradicating HPV related malignancy have had variable uptake and even where implementation has been successful the impact on HPV-OPC is not expected to be seen for decades.

Persistent oral high-risk HPV (hrHPV) is a risk factor for the development of HPV-OPC. The most common hrHPV types found in the western world are -16,-18,-31,-33 and -35 [1]. Predictors for oral hrHPV long term persistence are emerging from a growing literature. Key factors include age, male sex, immunosuppression, and a high HPV DNA viral load. In the cervix the persistence of hrHPV is used as a screening tool for cervical malignancy. No such screening process exists for persistent oral hrHPV and HPV-OPC. In a nested case control study Agalliu et al. [2] demonstrated that HPV-16 detection in saliva samples preceded incidence of clinical OPC by an average of 3.9 years. This and much of the recent literature, informs us of a critical fact – there is a long latent phase of hrHPV infection prior to development of clinically apparent malignancy. This provides us with a potential window for intervention prior to the development of advanced disease.

One of the principal barriers to developing a strategy for HPV-OPC screening is our lack of understanding of the natural history of the disease and in particular the risk and time course of progression from persistent hrHPV infection to malignancy. Unlike the cervix, HPV-OPC does not seem to follow a stepwise pattern of dysplasia, intraepithelial neoplasia, and invasive malignancy. The tonsillar crypts lack a basement membrane and an insitu form of the cancer is purported not to exist. Additionally, the oropharynx is an anatomically extensive region, making assessment and directed tissue sampling very difficult. This raises concerns regarding our ability to identify those in whom the disease is progressing from persistent hrHPV infection to HPV-OPC and by doing so, limiting further intervention to these specific individuals. Without this discrimination, screening will either lack sufficient sensitivity, or alternatively sufficient specificity, and we will risk over-treatment for those in whom the infection would have naturally cleared without progression, or fail to detect significant disease frequently leaving patients unassured. One emerging possibility to bypass this difficulty may be analysis of the temporal pattern of HPV expression and in particular detection of a rapid increase in the levels of HPV DNA over time.

One limitation to our current understanding of the potential for screening for OPC has been the lack of prospective studies of HPV biomarkers that demonstrate a link between persistent hrHPV and incident malignancy. Last year we reported the first case of an individual diagnosed with HPV-OPC following serial saliva testing for HPV-16 DNA as part of a prospective, longitudinal study. We detected occult, p16 positive OPC in an asymptomatic individual where MRI gave a negative result [3, 4]. This case allowed us to assess the pattern of HPV DNA expression prior to OPC development. The individual in question had increasing levels of oral HPV16DNA from ~3 to ~1300 viral copies/50 ng DNA over the course of the study. After surgical excision of the clinically occult 2 mm tonsillar HPV-OPC, HPV DNA became undetectable in saliva. A further report of persistent oral HPV 16 infection with incident diagnosis of HPV-OPC also demonstrated a rise in oral HPV 16 from low to high intensity, prior to the diagnosis of an invasive malignancy [5].

In our study we demonstrated that oral HPV-16 DNA was able to detect a 2 mm malignancy. The lesion was undetectable both clinically and radiologically. Despite the relatively high association of oral HPV-16 DNA with HPV-OPC positive tumours [6], screening high risk individuals may require a combination of saliva and serology to provide sufficient sensitivity and specificity. Both are highly acceptable low morbidity tests.

For screening to be beneficial, earlier diagnosis must provide patients with better clinical outcomes. HPV-OPC tends to present as locally advanced disease and despite having a favourable prognosis (10–20% 5-year recurrence rates), morbidity from treatment of advanced disease can be considerable, leaving some with lifelong pain, dysphagia, and speech issues. Those in whom the disease is found early can receive single modality treatment either through surgery or radiotherapy with excellent cure rates and lower side effects with virtually all patients achieving a normal diet and minimal long-term morbidity. Diagnosing the disease at an earlier stage would therefore appear desirable. However, given the low mortality of disease overall, this assertion remains controversial. Additionally, the difficulty in clinically diagnosing the disease at very early stages calls the feasibility of such an approach entirely into question, and it remains unclear as to how biomarker-positive patients should be clinically assessed, investigated and treated.

The apparent link between oral HPV copy number and progression from infection to microscopic disease is exciting and needs to be replicated on a larger scale. More accurate means for assessment of persistently HPV positive patients need to be established and new treatment options such as immunotherapeutic vaccines for clinically occult, very small volume disease need to be investigated. Recent findings help to inform us about both the natural history of this disease and the sensitivity and specificity of oral HPV DNA in detecting the disease. Rates of HPV-OPC are increasing rapidly. Further large-scale studies are the requirement for progressing our understanding of the feasibility of personalised screening in high-risk populations.
